# A Rare Case of Skin Necrosis Following Extravasation of Prothrombin Complex Concentrate (PCC) Infusion During Warfarin Reversal

**DOI:** 10.7759/cureus.37867

**Published:** 2023-04-20

**Authors:** Emma C Smith, Justin G Chen, Maha Bayya

**Affiliations:** 1 Internal Medicine, State University of New York (SUNY) Upstate Medical University, Syracuse, USA

**Keywords:** chemical burn, atrial fibrillation, anticoagulation, warfarin reversal, prothrombin complex concentrate, skin necrosis

## Abstract

Warfarin-induced skin necrosis is a well-documented complication that can occur following commencement of warfarin. However, skin necrosis following extravasation of prothrombin complex concentrate (PCC) infusion is a very rare adverse event that is not commonly documented. This case illustrates the possibility of developing skin necrosis following the administration of an anticoagulation reversal agent rather than from anticoagulation itself. We report a case of a 58-year-old male who developed skin necrosis at the site of PCC infusion in the right upper extremity (RUE) for warfarin reversal of an elevated international normalized ratio (INR). The skin necrosis progressed into a full thickness chemical burn. As a result, the patient underwent allograft followed by split thickness autograft and RECELL placement. This case presentation describes the first reported case of skin necrosis following extravasation of PCC infusion during warfarin reversal.

## Introduction

Patients on warfarin have very specific target ranges of international normalized ratio (INR), most being between 2.0 and 3.0 [1]. INR represents a way to measure the level of anticoagulation. Due to an increased bleeding risk on warfarin, patients have frequent INR monitoring to assess whether that is within their target ranges based on the specific indications for anticoagulation. Patients on warfarin spend up to 14.5%-19.3% of their time in supratherapeutic levels [1]. This is significant as the incidence of bleeding in patients on warfarin is approximately 15%-20% per year, with fatal cases of bleeding estimated at around 1%-3% per year [2]. Methods of management for elevated INR vary depending on four clinical scenarios: 1) INR > therapeutic range but < 4.5 with no significant bleeding; 2) INR > 4.5 but < 10.0 with no significant bleeding; 3) INR > 10.0 with no significant bleeding; and 4) life-threatening bleeding at any elevation of INR [2]. For an INR < 4.5 with no significant bleeding, management consists of lowering or omitting the warfarin dose and resuming once therapeutic range is achieved [1]. 

Guidelines for warfarin reversal in a patient with supratherapeutic INR and/or life-threatening bleeding from the 9th ed of the American College of Chest Physicians Evidence-Based Clinical Practice Guidelines are as referenced [3]. 

*Guideline 9.1*:

For patients taking Vitamin K antagonists (VKAs) with INRs between 4.5 and 10 and with no evidence of bleeding, we suggest against the routine use of Vitamin K (Grade 2B).

For patients taking VKAs with INRs > 10 and with no evidence of bleeding, we suggest that oral vitamin K be administered (Grade 2C).

*Guideline 9.3*: 

For patients with VKA-associated major bleeding, we suggest rapid reversal of anticoagulation with four-factor prothrombin complex concentrate (PCC) than with plasma (Grade 2C).

We suggest the additional use of vitamin K 5-10 mg administered by slow IV injection rather than reversal with coagulation factors alone (Grade 2C).

These guidelines should be referenced when a patient presents with supratherapeutic INRs so as to ensure the correct method of warfarin reversal is utilized. Administration of Vitamin K and PCC in the setting of warfarin reversal both come with potential side effects, like any medical intervention. Therefore, ensuring that administration is medically necessary according to guidelines is essential.

After extensive literature review, we believe that this case presentation describes the first reported case of full-thickness chemical burn following extravasation of PCC infusion in the setting of warfarin reversal for supratherapeutic INR.

## Case presentation

The patient is a 58-year-old male with a past medical history of atrial fibrillation and history of deep vein thromboembolism (DVT) on warfarin, hypertension, obstructive sleep apnea on continuous positive airway pressure (CPAP), obesity status-post gastric sleeve, and chronic venous stasis of bilateral lower extremities. The patient initially presented to Upstate University Hospital (UUH) at the request of the Upstate Coumadin Clinic where he was found to have a supra-therapeutic PT/INR of 90/11.7. When he presented to the emergency department at UUH, he complained of shortness of breath. He also admitted to four recent falls at home, with some of the falls having been accompanied by shortness of breath and lightheadedness. However, the most recent fall was mechanical and resulted in a large left gluteal hematoma. Upon presentation to the emergency department, imaging and repeat labs were done. Electrocardiogram (EKG) was done, along with chest X-ray, shoulder X-ray, pelvic X-ray, CT head, CT spine, CT abdomen/pelvis, and CT angiogram of chest along with doppler of lower extremities given history of fall and complaints of shortness of breath. Of note, computed tomography angiography (CTA) chest showed no evidence of pulmonary embolism (PE), and doppler ultrasound of lower extremities showed no evidence of DVT. His CT abdomen/pelvis showed large left gluteal hematoma 15 cm x 6.5 cm. EKG showed atrial fibrillation with right bundle branch block. Complete blood count (CBC) showed a hemoglobin/hematocrit (H/H) of 5.7/17.1 along with a repeat INR of > 11.78. The emergency department physicians decided to reverse the patient's warfarin due to patient's gluteal hematoma and elevated INR. The patient was given both phytonadione (Vitamin K) and PCC, and the patient's anticoagulation was held. Patient was also given two units of packed RBCs due to low H/H. The General Medicine team was consulted for the patient’s admission to the inpatient floors.

**Figure 1:**
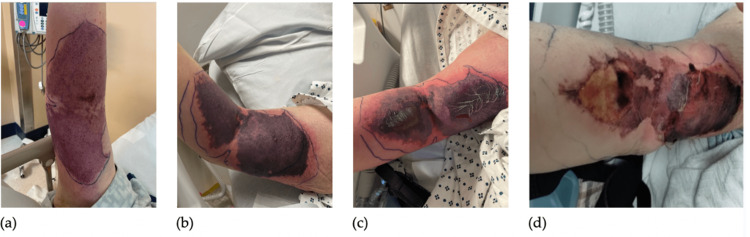
(a) RUE hours after PCC/Vitamin K infusion on 2/1/23. (b) RUE on day after infusion on 2/2/23. (c) RUE two days following infusion on 2/3/23. (d) RUE one week following infusion on 2/8/23. RUE, right upper extremity; PCC, prothrombin complex concentrate

Upon examination of the patient following his infusion of Vitamin K and PCC, the patient complained of severe pain in his right upper extremity (RUE) where the IV for the infusions was placed. The IV was subsequently removed and the area was erythematous and warm. Borders were drawn around the erythematous portion. On repeat examination, the erythema had transitioned to ecchymoses, still within the previously drawn borders (Figure 1a). It appeared to be the result of extravasation of the catheter site and thus of the PCC and Vitamin K infusion. The site progressed to tender purpura with surrounding erythema (Figure 1b). Two flaccid bullae appeared within the purpura (Figure 1c). Approximately one week later, the bullae ruptured, and Burns Surgery was consulted (Figure 1d).

**Figure 2:**
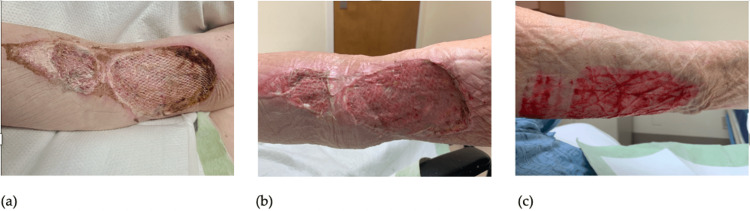
(a) RUE on 2/21/23 with placement of the allograft, taken prior to re-excision and placement of autograft. (b) RUE on 3/3/23, 7 days post-op from autograft taken from posterior RUE. (c) Donor graft site of right upper extremity on day 7 post-op, 3/3/23. RUE, right upper extremity

Burns surgery diagnosed the patient with a 1.5% total body surface area (TBSA) full thickness chemical burn secondary to the Vitamin K/PCC infusion. They recommended going to the operating room (OR) for burn debridement and excision with allografting. In the meantime, the wound was treated with Dakin’s soak and dressings. Unrelated to the RUE chemical burn, the patient also had a chronic wound that opened up and produced drainage on the left lateral thigh. Wound cultures grew Escherichia coli and blood cultures were obtained that grew Methicillin-resistant Staphylococcus aureus (MRSA). The patient began treatment with ceftriaxone and vancomycin. Ceftriaxone was later stopped by Infectious Disease and vancomycin was to be continued for 2 weeks pending negative repeat blood cultures. Despite positive blood cultures, the patient was taken to the OR on 2/12/23 where a debridement and excision and allograft placement was completed (Figure 2a). The plan was to take the patient back into the OR five days postoperatively for re-excision and autograft placement. Unfortunately, the patient began to experience episodes of shortness of breath and lightheadedness when ambulating, similar to how he described the episodes that preceded some of his falls at home. Cardiology was consulted, and after repeated EKGs and reviewing telemetry, it was found that the patient experienced paroxysmal third degree atrioventricular (AV) block. Anesthesiology was not comfortable with taking the patient back into the OR for re-excision until a pacemaker was placed. Burns noted that the allograft could remain in place for three weeks. Electrophysiology recommended pacemaker placement but had to wait 2 weeks as the patient was positive for MRSA bacteremia.

Leadless pacemaker placement was completed on 2/23/23 with subsequent re-excision of allograft with placement of autograft the next day. The donor site for the autograft was taken from the patient’s posterior right arm. On 2/24/23 repeat blood cultures (14 days after MRSA bacteremia diagnosis) came back negative. Through discussions with the patient throughout his stay, the patient has been switched from warfarin to apixaban due to the decreased risk of bleeding. The patient was discharged from the hospital to a short-term rehabilitation facility with appointments to follow-up with Burns as an outpatient. The patient was seen at the outpatient clinic for Burns surgery on day 7 post-op. On post-op evaluations, graft (Figure 2b) and donor sites (Figure 2c) were healing well.

## Discussion

Side-effects of PCC can vary from relatively benign symptoms such as headache, nausea, and vomiting to more fatal complications such as pulmonary embolism, deep vein thrombosis, and cerebrovascular accident [4]. A rare complication that has been noted in the literature is warfarin-induced skin necrosis (WISN) following recommencement of warfarin after use of an anticoagulation reversal agent, which in these two particular cases was Prothrombinex-VF [5-6]. However, transient hypercoagulability in these cases was hypothesized to have been likely due to Prothrombinex-VF containing concentrate of factors II, IX, and X and low levels of factors V and VII but not proteins C or S [5]. Our patient was given a four-factor PCC infusion which contains factor II, VII, IX, and X along with protein C and protein S [7]. This is the first reported case of full-thickness chemical burn and skin necrosis following extravasation of PCC without the recommencement of warfarin.

The presentation of WISN involves a sudden and localized painful skin lesion that typically manifests 3-10 days after commencement of warfarin. It presents as erythematous and subsequently become bullous, eventually progressing into gangrenous necrosis. This typically occurs in areas with increased subcutaneous fat, such as the abdomen and the thighs [5-6]. Our case presented in a very similar manner with an initial erythematous lesion that progressed to flaccid bullae which eventually ruptured. However, these lesions presented within 24 h in the RUE at the site of PCC infusion rather than an area with increased subcutaneous fat.

Associations of PCC infusion with increased thromboembolic risk has been well-documented in the literature, especially in warfarin reversal [8]. Patients who were already on an anticoagulant for a prothrombotic condition were linked with a higher risk of developing a thromboembolic complication post-PCC infusion [9]. Despite this association, the literature suggests that the increased thrombotic risk post-emergency PCC infusion may be due to a withdrawal of anticoagulation and underlying hypercoagulable conditions rather than the administration of PCC itself [7]. The patient had a history of atrial fibrillation and DVT on warfarin. The compound effects of stopping of warfarin at the time of PCC infusion may have left the patient in a relatively hypercoagulable state, thus resulting in thrombotic complications in the microvasculature of the skin where extravasation occurred [6]. 

The mechanism of how PCC may induce thromboembolic complications has not met a complete consensus in the literature yet. However, it is clear that a risk does exist, especially as PCC has a higher risk of thrombotic events compared to that of other warfarin reversal agents such as fresh frozen plasma (FFP) [8]. In all, it should be highlighted that skin necrosis can be a consequence of PCC infusion alone. It is paramount to recognize traditional risk factors of thrombotic complications, some of which include atrial fibrillation, obesity, and smoking. Accounting for such is crucial to minimize adverse events of PCC infusion and improve patient outcome.

## Conclusions

Skin necrosis following PCC infusion is a rare phenomenon that is currently not well documented in the literature. Familiarity with anticoagulation reversal guidelines is essential in optimizing patient outcomes. Potential adverse events of PCC infusion, which in our case was skin necrosis, should be noted, especially in patients with hypercoagulable conditions. Constant reassessment of the patient’s physical condition during anticoagulant reversal is paramount for prompt medical management of such potential sequelae.

## References

[1] Thigpen JL, Limdi NA (2013). Reversal of oral anticoagulation. Pharmacotherapy.

[2] Yee J, Kaide CG (2019). Emergency reversal of anticoagulation. West J Emerg Med.

[3] Holbrook A, Schulman S, Witt DM (2012). Evidence-based management of anticoagulant therapy: antithrombotic therapy and prevention of thrombosis, 9th ed: American College of Chest Physicians evidence-based clinical practice guidelines. Chest.

[4] Tabet R, Shammaa Y, Karam B, Yacoub H, Lafferty J (2018). Prothrombin complex concentrate and fatal thrombotic adverse events: a complication to keep in mind. Drug Discov Ther.

[5] Zhang L, Truong K, Chan L, Kim J, Fernandez-Peñas P (2022). Warfarin-induced skin necrosis after the use of an anticoagulation reversal agent. Australas J Dermatol.

[6] O'Dempsey RM, Choong AM, Patel A, Miller J, Walker PJ, Kruger A (2015). Warfarin-induced skin necrosis following recommencement of warfarin after perioperative Prothrombinex-VF. Med J Aust.

[7] Barco S, Picchi C, Trinchero A, Middeldorp S, Coppens M (2017). Safety of prothrombin complex concentrate in healthy subjects. Br J Haematol.

[8] Maguire M, Fuh L, Goldstein JN (2019). Thromboembolic risk of 4-factor prothrombin complex concentrate versus fresh frozen plasma for urgent warfarin reversal in the emergency department. West J Emerg Med.

[9] Owen EJ, Gibson GA, Human T, Wolfe R (2021). Thromboembolic complications after receipt of prothrombin complex concentrate. Hosp Pharm.

